# Deep Learning-Based Virtual Screening Identifies Potential Small-Molecule Inhibitors of ASK1: Natural Product Lead Discovery for MASH

**DOI:** 10.3390/ijms27146438

**Published:** 2026-07-20

**Authors:** Ruiqi Zhao, Jiahua Yang, Mengyao Han, Shiqi Tang, Hui Hu, Jiesheng Guo, Mengqing Ma, Jialing Sun, Xiaozhou Zhou

**Affiliations:** 1 The Fourth Clinical Medical College, Guangzhou University of Chinese Medicine, Shenzhen 518033, China; 20252120304@stu.gzucm.edu.cn (R.Z.);; 2 The Second Clinical Medical College, Guangzhou University of Chinese Medicine, Guangzhou 510006, China; 3 Faculty of Chinese Medicine, Macau University of Science and Technology, Taipa, Macao 999078, China

**Keywords:** metabolic dysfunction-associated steatohepatitis, ASK1, natural products, virtual screening, molecular dynamics simulation, MM-GBSA, density functional theory

## Abstract

Apoptosis signal-regulating kinase 1 (ASK1) represents a critical therapeutic target for metabolic dysfunction-associated steatohepatitis (MASH). Natural products, owing to their unique chemical diversity, constitute a rich reservoir for discovering novel ASK1 inhibitors. The emergence of artificial intelligence-assisted drug discovery (AIDD) has opened new avenues for exploring small-molecule inhibitors. Through virtual screening, molecular docking, interaction profiling, molecular dynamics simulations, and MM-GBSA binding free energy calculations, we systematically evaluated the binding mode, stability, and key residue contributions of the CMNPD10921–ASK1 complex. CMNPD10921 stably occupied the ASK1 active pocket, forming hydrophobic interactions and hydrogen bonds with multiple key amino acid residues. MM-GBSA analysis yielded a total computed binding free energy of −31.15 kcal/mol, suggesting a computationally favorable interaction, with van der Waals forces serving as the dominant energetic driver of complex stabilization. Residue energy decomposition further identified ILE324, THR288, and THR639 as major contributors to ligand binding. Integrating deep learning, molecular simulation, and quantum chemical calculations, this study successfully identified CMNPD10921 from a vast natural product library as a putative lead compound candidate targeting the ASK1 central regulatory region, offering a novel candidate molecule for anti-MASH drug development.

## 1. Introduction

Nonalcoholic fatty liver disease has long been recognized as one of the most prevalent chronic liver disease spectra worldwide. With the escalating prevalence of metabolic disorders, obesity, and type 2 diabetes, the disease burden continues to expand rapidly. In recent years, international consensus has updated the traditional NAFLD/NASH nomenclature to metabolic dysfunction-associated fatty liver disease (MAFLD) and metabolic dysfunction-associated steatohepatitis (MASH), better reflecting the metabolic pathogenesis of the disease [1,2]. MASH is characterized not only by hepatic steatosis, ballooning degeneration, and lobular inflammation but also, more critically, by the progression of liver fibrosis. Fibrosis stage has been robustly associated with liver-related events, liver transplantation requirements, and all-cause mortality [3,4,5]. Thus, interrupting hepatocellular stress injury, inflammatory amplification, and fibrotic deposition lies at the core of MASH therapeutic strategies.

Current MASH drug development is advancing rapidly. The thyroid hormone receptor β agonist resmetirom has demonstrated the ability to improve histologic endpoints in phase III trials; GLP-1 receptor agonists, glucagon-like peptide-based multitarget agonists, and pan-PPAR agonists have also shown varying degrees of metabolic and histologic benefits in clinical studies [6,7,8,9]. Nevertheless, MASH exhibits substantial heterogeneity, and a single metabolic intervention may not adequately cover the pathological dimensions of inflammation, cell death, and fibrotic progression. Therefore, anti-inflammatory and anti-fibrotic targets addressing stress signaling, inflammatory transduction, and hepatic stellate cell activation remain essential components of novel MASH therapeutic regimens [10].

Apoptosis signal-regulating kinase 1 (ASK1) is a stress-responsive mitogen-activated protein kinase kinase kinase (MAP3K) that can be activated by diverse pathological stimuli, including oxidative stress, lipotoxicity, endoplasmic reticulum stress, inflammatory cytokines, and mitochondrial dysfunction. Upon activation, ASK1 phosphorylates MKK4/7 and MKK3/6, thereby initiating the JNK and p38 MAPK signaling pathways, which in turn regulate apoptosis, inflammatory cytokine expression, hepatic stellate cell activation, and extracellular matrix deposition. Given that oxidative stress and lipotoxic injury permeate multiple stages of MASH pathogenesis, ASK1 has been regarded as a pivotal hub linking metabolic stress to fibrotic progression [11]. Inhibition of ASK1 has been shown to ameliorate inflammation and fibrosis in animal models of MASH [12,13]. Importantly, ASK1-knockout mice exhibit no overt phenotype under basal conditions [14], supporting ASK1 inhibition as a potentially safe therapeutic strategy. Multiple small-molecule ASK1 inhibitors have been developed and evaluated in preclinical and clinical settings. Selonsertib, a potent and selective ATP-competitive ASK1 inhibitor, demonstrated promising antifibrotic effects in preclinical NASH models and advanced to clinical trials in patients with advanced fibrosis due to NASH [11]. NQDI-1, a quino-line-3-carboxamide derivative, represents another class of ASK1 inhibitors that has shown efficacy in preclinical models of neurodegeneration and inflammatory diseases [15]. K811, a selective ASK1 inhibitor, has demonstrated protective effects in models of gastric cancer [16]. Despite these advances, no ASK1 inhibitor has yet achieved regulatory approval, highlighting the continued need for structurally diverse candidates with optimized properties, potentially including those derived from natural product scaffolds. Hence, the discovery of ASK1 candidate molecules featuring novel chemical scaffolds, distinct binding modes, and optimizable pharmacokinetic properties holds considerable promise.

Natural products have historically served as a vital source for drug discovery, offering structural novelty, stereochemical complexity, and bioactivity diversity that synthetic compound libraries often cannot rival [17,18]. Marine natural products, owing to their unique ecological habitats, frequently harbor skeletal types and functional group combinations rarely found in terrestrial natural products, rendering them an important resource for anti-cancer, anti-infective, anti-inflammatory, and metabolic disease drug development [19]. With the continuous development of open-access databases, resources such as COCONUT (COlleCtion of Open Natural prodUcTs) [20] and CMNPD (Comprehensive Marine Natural Products Database) [21] now provide systematic chemical space for large-scale virtual screening of natural products. COCONUT integrates extensive open-source natural product structural information, while CMNPD focuses on marine natural products with biological source and activity annotations, furnishing a data foundation for discovering novel natural product ligands targeting specific proteins.

Concurrently, computer-aided drug design and AI-assisted drug discovery are significantly transforming early-stage drug development pipelines. Structure-based virtual screening, deep learning-based conformational prediction, molecular dynamics simulations, and end-point free energy calculations can rapidly narrow the candidate space prior to experimental screening, providing complementary evidence on binding conformation, thermodynamic contributions, and dynamic stability [22,23]. Particularly for large, structurally complex, and experimentally costly compound collections such as natural product libraries, computational screening facilitates the prioritization of lead molecules with plausible binding modes and optimizable potential.

Against this backdrop, the present study integrates deep learning-based pocket prediction, natural product drug-likeness filtering, DiffDock high-throughput virtual screening, QVina2 molecular docking cross-validation, PLIP interaction analysis, molecular dynamics simulations, MM-GBSA binding free energy calculations, and DFT electronic structure analysis to systematically screen and evaluate potential small-molecule inhibitors of ASK1, thereby providing a renewed computational basis for the development of anti-inflammatory and anti-fibrotic MASH therapeutics.

## 2. Results

### 2.1. Binding Pocket Identification

**Figure 1:**
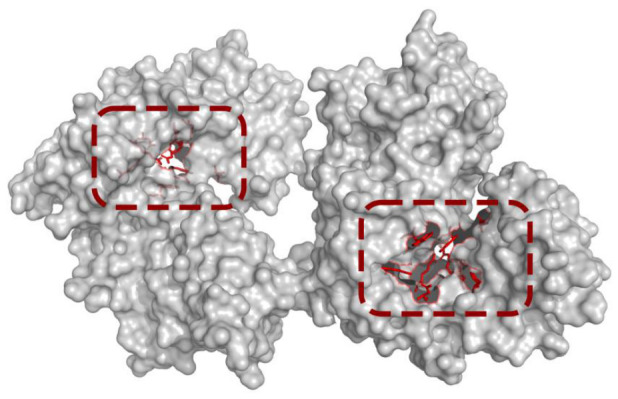
UniSite-predicted ligand-binding pockets of ASK1. The highest-scoring binding pocket selected for virtual screening is displayed, with red-highlighted surface regions indicating the predicted ligand-binding site. The two spatially separated red regions enclosed within the boxes correspond to the same predicted binding pocket located symmetrically on each protomer of the ASK1 homodimer.

The UniSite model identified 11 potential binding pockets on ASK1, which differed in spatial distribution, volume, and predicted drug score (Table S1). To ensure accuracy and biological relevance of subsequent virtual screening, we selected the highest-confidence pocket with a drug score ≥ 0.8 as the target site for all docking studies. Figure 1 visualizes this selected binding pocket, with red regions indicating the predicted binding site. This pocket is located in a deep interdomain cleft within the ASK1 central regulatory region, consistent with typical small-molecule binding cavity characteristics. Complete geometric and scoring information for all eleven predicted pockets is provided in Table S1.

### 2.2. Virtual Database Construction

Drug-likeness assessment was conducted on the initial natural product ligand library. Approximately 700,000 small molecules underwent a preliminary virtual filtering process guided by Lipinski’s Rule of Five and Quantitative Estimate of Druglikeness (QED) score criteria, resulting in the identification of 37,280 compounds exhibiting favorable drug candidacy from the COCONUT database and 47,450 compounds from the marine natural product subset. The resulting collection of 84,730 compounds, initially represented as one-dimensional SMILES strings, underwent three-dimensional (3D) structure generation using the cheminformatics toolkit RDKit. Preprocessing of all 3D molecular structures was carried out with AutoDock Tools, which included polar hydrogen atom incorporation and charge computation, before final conversion to PDBQT format suitable for molecular docking procedures.

### 2.3. AI-Assisted Drug Screening

Using DiffDock (version 1.1.3), we performed deep learning-based protein–ligand binding assessments for the target protein against the 37,280 ligands from COCONUT and the 47,450 ligands from CMNPD. Binding capacity was evaluated by the confidence score: values below −1.5 indicated ineffective protein–ligand interactions; values between −1.5 and 0 indicated feasible binding potential; and values above 0 indicated stable binding complexes. From the COCONUT database, 6505 candidate compounds were selected, among which 48 exhibited confidence scores greater than 0 (Table S2); from the CMNPD, 6338 candidate compounds were selected, with 33 showing confidence scores above 0 (Table S3). To further prioritize candidates for computationally intensive downstream validation, we applied an additional stringency threshold, selecting only compounds with confidence scores exceeding 20. This threshold was empirically determined based on the score distribution to focus computational resources on the highest-ranking predictions from the deep learning model, yielding a manageable set of top-tier candidates for detailed multi-method validation. Compounds meeting this criterion are summarized in Table 1.

### 2.4. Molecular Docking

These molecules were subjected to molecular docking using QVina2, and binding energies (BE) were calculated (Table 2). Negative BE values indicate computationally favorable predicted protein–ligand interactions; more negative values suggest stronger predicted binding propensity based on the force field scoring function. Conversely, values near or above zero reflect weak or unfavorable predicted interactions. After stringent selection, CMNPD10921 exhibited the most negative binding energy with ASK1, demonstrating excellent predicted binding affinity, and was therefore designated as the core candidate compound for further validation through molecular dynamics simulations and binding free energy calculations.

### 2.5. Interaction Mode Analysis

**Figure 2:**
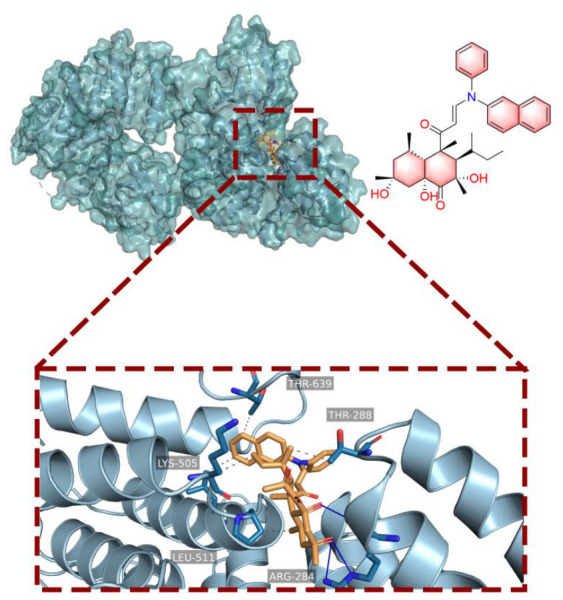
Non-covalent interaction profiles of CMNPD10921 with ASK1. Molecular interactions analyzed by PLIP and visualized in PyMOL. The upper panel shows the molecular surface representation of the ASK1 binding pocket with the docked CMNPD10921 ligand. The lower panel provides a detailed view of protein–ligand interactions. The 2D chemical structure of CMNPD10921 is shown on the right. Ligands and key residues are depicted as stick models; hydrogen bonds (blue lines) and hydrophobic interactions (gray dashed lines) are indicated.

The PLIP tool was employed to achieve precise characterization of the binding architecture and pharmacophoric attributes of the primary ligand bound to the target protein through exhaustive evaluation of non-covalent interactions in the optimal docking configuration. Leveraging the PLIP algorithm, we performed systematic detection and quantification of diverse interaction categories, including hydrogen bond patterns, hydrophobic associations, electrostatic contacts, and π–π stacking interactions established between the ligand and the receptor’s binding cavity. An interactive interface visualization presented the ligand and essential interacting residues as stick representations, with hydrogen bond spatial orientations clearly depicted. Additionally, the electrostatic potential surface of the receptor was constructed to illustrate the docking relationship. The analysis revealed that CMNPD10921 formed five hydrophobic interactions and three hydrogen bonds with five key ASK1 residues: THR288, THR639, LYS505, LEU511, and ARG284 (Figure 2).

These results indicate that CMNPD10921 and ASK1 are capable of forming a relatively stable docking complex.

### 2.6. Molecular Dynamics Simulations

**Figure 3:**
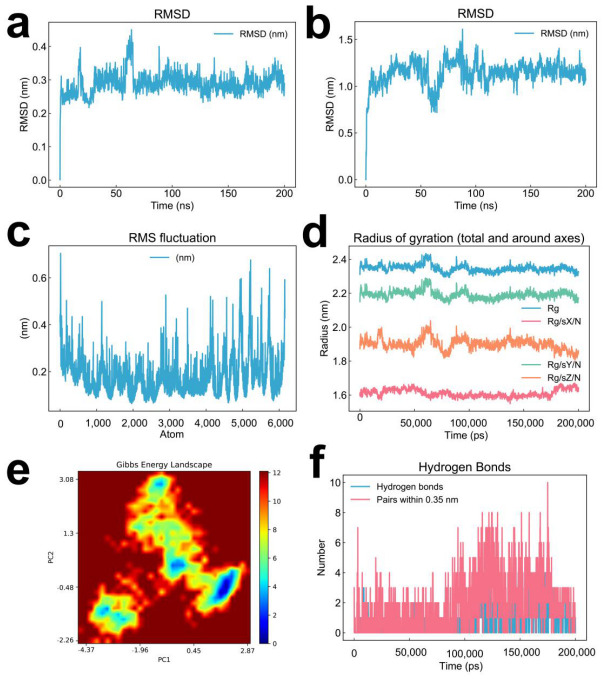
Structural stability metrics from 200 ns molecular dynamics simulations. (**a**,**b**) Root-mean-square deviation (RMSD) of protein backbone and ligand atoms over time for: (**a**) ASK1 backbone and (**b**) ligand in ASK1 complex. (**c**) Root-mean-square fluctuation (RMSF) per residue. (**d**) Radius of gyration (Rg) over simulation time. (**e**) Gibbs free energy landscapes projected onto principal components PC1 and PC2. (**f**) Time evolution of intermolecular hydrogen bonds.

Based on the above screening pipeline, we identified CMNPD10921 as a potential lead compound targeting ASK1. To further validate the stability of its binding, we performed 200 ns molecular dynamics simulations. From the root-mean-square deviation (RMSD) profiles, both the protein backbone (Figure 3a) and the ligand (Figure 3b) of the ASK1–CMNPD10921 complex exhibited strong stability in the later stages of simulation, indicating that the protein–ligand complex maintained good structural integrity and binding stability throughout the simulation. The protein did not undergo large-scale conformational changes, and the ligand remained persistently bound within the pocket, providing reliable conformations for subsequent binding free energy calculations.

Root-mean-square fluctuation (RMSF) reflects the positional fluctuation of each residue during the simulation. High RMSF values typically correspond to flexible loop regions or solvent-exposed loops, whereas low RMSF values correspond to core structural domains or secondary structure elements. RMSF analysis indicated that in the ASK1–CMNPD10921 system (Figure 3c), residues and the small molecule exhibited minimal fluctuations, suggesting that the complex maintained relatively high structural stability throughout the simulation.

The radius of gyration (Rg) measures the distribution of protein mass relative to its centroid and serves as a sensitive indicator of overall protein compactness and folding state. For well-folded globular proteins, Rg values should remain stable within a range consistent with molecular weight; a sudden increase in Rg typically indicates protein unfolding or domain dissociation. In the ASK1 system, upon ligand binding (Figure 3d), Rg values showed minimal variation, confirming that the protein maintained a compact folded state with strong structural stability.

Two-dimensional Gibbs free energy landscape (FEL) analysis based on principal component analysis (PCA) provided an overview of the energy distribution of the complex along major conformational modes. This analysis projected the high-dimensional conformational space onto a two-dimensional plane (Figure 3e), illustrating energy landscapes under different principal components (PCs). Blue regions indicate lower energy states of the ligand–protein complex, corresponding to more stable binding. The system exhibited a single or adjacent blue energy basin with secondary energy minima, indicating the presence of multiple predominant conformational states during the simulation—consistent with the homodimeric structure of ASK1. Hydrogen bond analysis further showed that the ASK1–CMNPD10921 system (Figure 3f) maintained a stable number of hydrogen bonds after 100 ns of simulation.

### 2.7. Computation of Binding Free Energy

**Figure 4:**
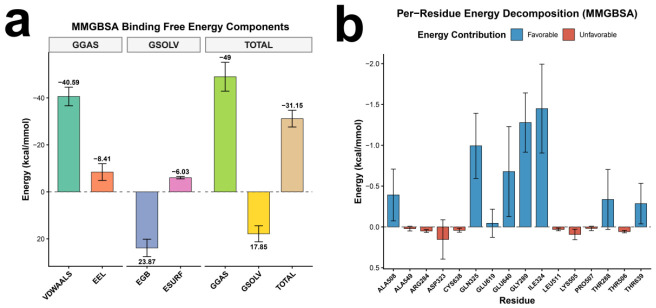
MM-GBSA binding free energy decomposition. (**a**) Energy contribution profiles showing van der Waals (ΔVDWAALS), electrostatic (ΔEEL), polar solvation (ΔEGB), and nonpolar solvation (ΔESURF). (**b**) Bar chart of the energy contribution of each residue.

For a comprehensive assessment of the candidate compound’s binding affinity, the MM-GBSA methodology was applied to compute the binding free energy for the ASK1–CMNPD10921 complex, accompanied by energy decomposition analysis (Figure 4a). Energy decomposition demonstrated that van der Waals interactions (ΔVDWAALS) constituted the predominant thermodynamic contributor to ligand–protein association, with a value of −40.59 kcal/mol. This indicates excellent geometric complementarity between the candidate molecule and the target’s binding cavity. While electrostatic interactions (ΔEEL) provided favorable contributions under gas-phase conditions (−8.41 kcal/mol), these were largely counterbalanced by polar desolvation penalties (ΔEGB = +23.87 kcal/mol), yielding a net unfavorable polar term—a characteristic thermodynamic pattern observed in typical protein–ligand complexes. The non-polar solvation component (ΔESURF = −6.03 kcal/mol) contributed modestly but favorably through hydrophobic effects. The calculated total binding free energy of CMNPD10921 with ASK1 was −31.15 kcal/mol. While this computationally estimated value suggests potentially favorable binding, it should not be directly equated with experimentally measured binding affinity, and the magnitude of this figure is best interpreted as an indicator of relative binding favorability rather than a quantitative prediction of potency.

Furthermore, residue-wise energy decomposition (Figure 4b) indicated that ALA508, GLN325, GLU619, GLU640, GLY289, ILE324, THR288, and THR639 contributed favorably to CMNPD10921 binding to ASK1, with ILE324 providing the largest contribution. Conversely, ALA549, ARG284, ASP323, CYS638, LEU511, LYS505, PRO507, and THR506 exhibited unfavorable energetic contributions.

### 2.8. Electronic Structure Evaluation by DFT

**Figure 5:**
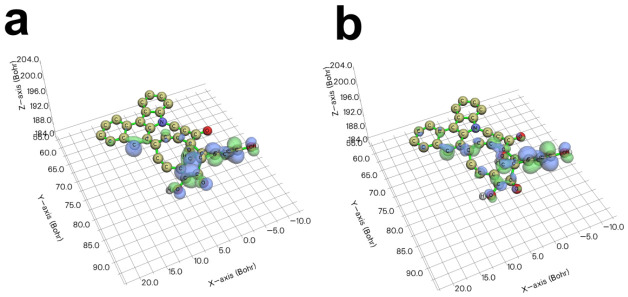
Frontier molecular orbital distributions of CMNPD10921 from DFT calculations. Displayed spatial distributions of: (**a**) LUMO (−4.630266 eV) and (**b**) HOMO (−5.630530 eV). HOMO-LUMO energy gap: 1.000264 eV.

For examination of the chemical reactivity profile and drug-likeness characteristics of the candidate compound at the electronic level, we applied density functional theory (DFT) methodology to CMNPD10921, concentrating on both the energetic and spatial attributes of frontier molecular orbitals (FMOs). Frontier molecular orbital characterization indicated HOMO and LUMO energies of −5.630530 eV and −4.630266 eV, respectively, corresponding to a HOMO–LUMO energy gap (ΔE) of 1.000264 eV. This moderately low gap indicates that the molecule possesses favorable electronic responsiveness and moderate polarizability, conducive to electronic redistribution upon binding with the target protein, thereby enhancing interaction stability. Regarding spatial orbital distribution, the LUMO electron distribution (Figure 5a) differed subtly from that of the HOMO (Figure 5a), suggesting that these regions may act as electron acceptors in stabilizing interactions during binding. However, while the relatively small ΔE favors enhanced electronic adaptability, it also implies potential susceptibility to oxidative metabolism in vivo.

## 3. Discussion

This study established a multi-tiered screening pipeline centered on the stress-signaling target ASK1 for anti-inflammatory and anti-fibrotic MASH therapy. The pipeline integrated deep learning-based pocket prediction, natural product library filtering, AI-assisted docking, traditional molecular docking cross-validation, interaction profiling, molecular dynamics simulations, MM-GBSA binding free energy calculations, and DFT electronic structure analysis. The results revealed that CMNPD10921, derived from the CMNPD marine natural product database, exhibited consistent lead-like characteristics across multiple computational dimensions: it stably occupied the predicted ASK1 binding pocket, formed multi-point interactions with THR288, THR639, LYS505, LEU511, and ARG284; 200 ns molecular dynamics simulations supported favorable conformational stability of the complex; MM-GBSA calculations yielded a total binding free energy of −31.15 kcal/mol, with van der Waals and nonpolar solvation forces as the primary favorable contributors; and DFT analysis further indicated strong electronic responsiveness and moderate polarization adaptability. Collectively, these findings support CMNPD10921 as a potential small-molecule inhibitor of ASK1, warranting further experimental validation.

CMNPD10921, with the chemical name 10-hydroxy-18-N-2-naphthyl-N-phenylaminobetaenone, molecular formula C_37_H_45_NO_5_, and a relative molecular mass of approximately 583.77 g/mol, features a large hydrophobic carbon skeleton containing naphthyl, phenyl, carbonyl, hydroxyl, and tertiary amine moieties. This structure exhibits coexisting aromaticity, hydrophobicity, and polar functional groups. The naphthyl and phenyl groups may enhance π–π stacking or hydrophobic contacts with the protein’s hydrophobic pocket, while multiple hydroxyl and carbonyl groups offer potential sites for hydrogen bonding or polar interactions. Concurrently, the elongated lipophilic skeleton facilitates deep insertion into the binding pocket with favorable shape complementarity, consistent with the dominant role of van der Waals interactions observed in MM-GBSA results. Thus, stable binding of CMNPD10921 to ASK1 likely results from a combination of hydrophobic contacts, aromatic ring interactions, van der Waals forces, and local polar interactions, rather than relying solely on hydrogen bonding.

ASK1 serves as a critical signaling node connecting oxidative stress, inflammatory amplification, and cell death. Previous studies have shown that the ASK1 inhibitor selonsertib exhibited trends toward fibrosis improvement in early MASH clinical trials [11]. Multiple mechanistic studies continue to support the involvement of ASK1 in hepatic stellate cell activation, cell death, mitochondrial damage, and inflammatory signal transduction [12,24,25,26]. Conversely, some studies suggest that hepatocyte ASK1 may exert protective effects in certain NAFLD models, with its deletion paradoxically exacerbating steatosis, inflammation, and fibrosis [27]. These seemingly contradictory findings indicate that ASK1 function in liver disease may be cell-type dependent, injury-mode dependent, and disease-stage dependent. Therefore, subsequent investigations of CMNPD10921 should not be confined to enzyme-target binding validation but should further delineate its functional effects in hepatocytes, hepatic stellate cells, Kupffer cells, and macrophages and confirm its genuine signal-inhibitory activity by monitoring downstream markers such as p-ASK1, p-MKK, p-JNK, and p-p38.

In addition, our use of the ASK1 central regulatory region structure (PDB: 5ULM) rather than the kinase catalytic domain represents a deliberate strategy to explore allosteric modulation mechanisms. ASK1 activity is regulated through multiple non-catalytic mechanisms, including oxidative modification of cysteine residues, protein–protein interactions, and conformational changes within the central regulatory region, which contains functionally critical coiled-coil domains for oligomerization and interfaces for regulatory protein binding. Allosteric modulation offers theoretical advantages over ATP-competitive inhibition, including improved selectivity due to greater structural divergence of regulatory regions across the kinome, accommodation of structurally diverse ligands not constrained by ATP-mimicry requirements, and potential for more nuanced functional regulation rather than complete catalytic blockade.

From a binding mode perspective, the interaction between CMNPD10921 and ASK1 is predominantly hydrophobic in nature. PLIP analysis identified five hydrophobic interactions and three hydrogen bonds with THR288, THR639, LYS505, LEU511, and ARG284. MM-GBSA energy decomposition further showed that the total binding free energy was primarily contributed by van der Waals forces, with ΔVDWAALS reaching −40.59 kcal/mol, indicating favorable spatial complementarity and hydrophobic surface matching between the candidate molecule and the ASK1 pocket. This characteristic is consistent with the large hydrophobic aromatic fragments and polycyclic framework of CMNPD10921. For kinase targets, stable hydrophobic embedding, localized hydrogen bond anchoring, and pocket shape complementarity collectively determine ligand residence time and binding stability. In this study, both THR288 and THR639 were identified as interacting residues by PLIP and also exhibited favorable contributions in MM-GBSA residue decomposition, suggesting that they may serve as key anchoring residues maintaining the stability of the ASK1–CMNPD10921 complex. While MM-GBSA calculations provide valuable insights into the energetic basis of protein–ligand interactions, several inherent limitations must be acknowledged. These include force field approximations that cannot fully capture polarization effects or charge transfer, implicit solvation models that approximate explicit water and ion environments, single-trajectory approaches that may underestimate entropic penalties, and systematic errors that can lead to overestimation of binding free energy magnitudes—the calculated value of −31.15 kcal/mol should be interpreted primarily for relative compound ranking rather than as a quantitative prediction of absolute binding affinity.

It is important to emphasize that discrepancies between PLIP geometric interaction analysis and MM-GBSA residue energy decomposition are not only expected but methodologically reasonable. PLIP primarily identifies hydrogen bonds, hydrophobic contacts, and π-related interactions based on distance, angle, and contact type criteria in static or representative conformations. In contrast, MM-GBSA residue decomposition estimates averaged energy terms—van der Waals, electrostatic, polar solvation, and nonpolar solvation—over trajectory snapshots [28,29,30]. Thus, a residue identified by PLIP as forming a hydrogen bond or hydrophobic contact may still present as energetically unfavorable in net decomposition due to polar desolvation penalties, local conformational strain, suboptimal contact geometry, side-chain flexibility loss, or solvent exposure. In this study, ARG284 formed three hydrogen bonds yet exhibited an unfavorable contribution in MM-GBSA, likely attributable to the high polar desolvation cost associated with the charged guanidinium group entering the binding interface. If the hydrogen bonds did not maintain ideal geometry throughout the dynamic trajectory, the electrostatic gain may have been insufficient to offset the solvation penalty. Similarly, LYS505 formed two hydrophobic interactions with CMNPD10921, and LEU511 formed one, yet both emerged as unfavorable residues in energy decomposition—potentially reflecting inadequate local packing, low contact occupancy, or a high conformational penalty induced upon binding. Conversely, ILE324, though not prominently highlighted as a key interacting residue in PLIP, contributed the most favorable energy in MM-GBSA, suggesting that it may exert a major contribution to total binding free energy through sustained, extensive hydrophobic complementarity that is not captured by a single geometric rule.

Molecular dynamics simulations further support the reliability of the CMNPD10921 binding conformation with ASK1. The protein backbone RMSD and ligand RMSD stabilized in the later stages of simulation, indicating that the complex did not undergo significant conformational disassembly or ligand egress. RMSFs remained modest overall, reflecting maintained stability in key residue regions. The minimal Rg variation suggests that global ASK1 compactness was not disrupted upon ligand binding. PCA-based free energy landscape analysis revealed a stable energy basin with secondary dominant conformational states, suggesting that the complex does not remain trapped in a single rigid conformation but instead dynamically adapts within a limited conformational space. Such limited flexibility may facilitate stable ligand retention within the kinase pocket while permitting local side-chain rearrangement. The hydrogen bond count remained relatively stable after 100 ns, dynamically supporting the potential involvement of polar residues such as ARG284 in the local binding network. It should be noted that MD simulations and MM-GBSA represent computational predictive evidence; their results are influenced by initial conformation, force field parameters, ligand charge fitting, protonation states, water molecule treatment, and sampling length and cannot be directly equated with experimental outcomes.

DFT calculations provided complementary insights into the electronic structure of CMNPD10921. The HOMO energy was −5.630530 eV, the LUMO energy was −4.630266 eV, and the HOMO–LUMO gap was 1.000264 eV. The relatively small gap suggests that the molecule possesses high electronic polarizability and favorable electron redistribution potential, which may facilitate adaptation to the local electrostatic environment within the binding pocket and enhance interactions with polar or aromatic residues. On the other hand, a small gap also indicates potential oxidative metabolic liability. Given the presence of a large hydrophobic aromatic system and polycyclic framework, subsequent optimization should prioritize the evaluation of solubility, microsomal stability, CYP-mediated metabolic risk, hERG potential, and nonspecific protein binding. For natural product lead compounds, strong binding affinity often accompanies high structural complexity. Future optimization strategies may include preserving key hydrophobic anchor regions and hydrogen bond donor/acceptor features, moderately reducing hydrophobic volume, optimizing polar surface area, and improving metabolic soft spots to enhance drug-likeness. While DFT analysis did not alter our selection of CMNPD10921 as the lead candidate, it provides a theoretical foundation for anticipating ADME challenges and rational design of optimized analogs with improved drug-like properties while retaining key pharmacophoric features responsible for ASK1 binding.

This study employed two natural product databases, COCONUT and CMNPD, as screening sources, leveraging the general chemical space of natural products alongside the unique skeletal resources of marine natural products. In recent years, the standardization and open-access nature of natural product databases have substantially enhanced the feasibility of structure-based virtual screening [17]. Deep learning-assisted docking can rapidly process large ligand libraries and offers advantages over traditional search algorithms in conformational generation. However, confidence scores should not be equated with binding free energy or biological activity. Therefore, we further employed QVina2, PLIP, MD, and MM-GBSA for multi-dimensional cross-validation to mitigate false positives arising from any single scoring function. Nevertheless, computational screening can only provide prioritization evidence. For CMNPD10921, the most critical validation path should include: (1) in vitro ASK1 kinase activity inhibition assays; (2) assessment of its inhibitory effect on ASK1-mediated JNK/p38 phosphorylation; (3) evaluation of its impact on cell death, oxidative stress, and inflammatory cytokine expression in lipotoxicity-induced hepatocyte injury models; (4) detection of α-SMA, COL1A1, TIMP1, and MMP-related markers in TGF-β or lipotoxicity-associated hepatic stellate cell activation models; and (5) assessment of NAS score, liver fibrosis area, hydroxyproline content, and serum liver enzymes in diet-induced or chemical–diet combined MASH animal models.

From the perspective of the MASH therapeutic landscape, future drug development may increasingly favor precision stratification and combination interventions. The clinical success of resmetirom has demonstrated the feasibility of targeting metabolic pathways to improve MASH histologic endpoints. GLP-1 receptor agonists, tirzepatide, and FGF21 analogs underscore the importance of weight loss, insulin resistance, and fat metabolism improvement [6,7,9]. However, for patients with established inflammation and fibrosis progression, metabolic improvement alone may be insufficient to reverse all pathological processes. Therefore, ASK1 inhibitors or ASK1 pathway modulators are more likely to serve as components of anti-inflammatory and anti-fibrotic strategies, complementing metabolic regulators. Given that previous ASK1 inhibitor clinical studies did not meet their primary endpoints, the development of CMNPD10921 should pay particular attention to patient stratification logic, target occupancy, hepatic tissue exposure, cell-type selectivity, and long-term safety evaluation.

While the individual computational tools employed in this study are well-established, several aspects distinguish this work from previous virtual screening efforts. First, this represents, to our knowledge, the first systematic exploration of large-scale marine natural product chemical space for ASK1 inhibitor discovery, leveraging the unique structural diversity and biosynthetic complexity of compounds from extreme ecological niches that may yield modulators with novel mechanisms of action. Second, our multi-layered cross-validation strategy integrates AI-assisted pose prediction, traditional molecular docking, extended molecular dynamics simulations, MM-GBSA calculations, and DFT analysis, with convergence across these orthogonal theoretical frameworks substantially reducing false positives and increasing confidence in the identified lead compound. Third, our systematic funnel approach—progressing from ~700,000 natural products through sequential filtering based on drug-likeness, AI-predicted binding confidence, docking affinity, interaction quality, and dynamic stability—represents a rigorous prioritization strategy particularly crucial for structurally complex natural product libraries, where computational costs are significantly higher than for conventional drug-like compounds.

It should be noted that the present study did not include direct computational comparisons with known ASK1 inhibitors such as selonsertib or NQDI-1. This decision reflects our study’s primary objective: to explore structurally novel natural product scaffolds with potentially distinct binding modes and mechanisms of action, rather than to optimize existing inhibitor chemotypes. Known ASK1 inhibitors were developed as ATP-competitive kinase inhibitors targeting the catalytic domain, whereas our screening focused on predicted binding pockets within the central regulatory region, aiming to identify potential allosteric modulators. However, it must be emphasized that all findings presented in this study are based on computational predictions, which, despite employing state-of-the-art methodologies and multi-method cross-validation, cannot substitute for experimental validation. Future work should prioritize: (1) in vitro binding confirmation through biophysical methods (SPR, MST, ITC, thermal shift assays); (2) enzymatic activity assays to determine whether CMNPD10921 inhibits ASK1 kinase activity or modulates its function through allosteric mechanisms; (3) cellular functional studies assessing effects on ASK1-mediated JNK/p38 signaling, lipotoxicity-induced apoptosis, oxidative stress responses, and inflammatory cytokine production in hepatocytes, as well as effects on hepatic stellate cell activation and fibrogenic marker expression; (4) pharmacokinetic and ADME profiling to evaluate oral bioavailability, metabolic stability, and potential liabilities predicted by DFT analysis; and (5) in vivo efficacy assessment in diet-induced or chemical–diet combined MASH models, measuring histologic endpoints (NAS score, fibrosis stage), biochemical markers, and mechanistic pathway indicators.

Overall, this study identified CMNPD10921 as a potential ASK1 inhibitor, demonstrating its stable binding conformation, favorable predicted binding free energy, and reasonable electronic structure basis. The most salient binding feature of this molecule is its van der Waals and hydrophobic force-driven interaction, supplemented by a localized hydrogen bond network maintaining conformational stability. Although this study remains at the computational prediction stage, lacking enzymatic, cellular, and animal experimental validation, its findings offer a new lead clue for the discovery of natural product-derived ASK1 inhibitors and lay a foundation for subsequent experimental investigation and structural optimization of anti-MASH, anti-inflammatory, and anti-fibrotic drug candidates.

## 4. Materials and Methods

### 4.1. Protein Target Structure Preparation

The three-dimensional coordinates of ASK1 were obtained from the Protein Data Bank (https://www.rcsb.org/, accessed on 6 May 2026) under the accession code pdb_00005ulm. Structural visualization and quality assessment were conducted using PyMOL (version 3.1.6.1). Following confirmation of protein chain completeness, all co-crystallized ligands and irrelevant heteroatoms were eliminated to generate a clean receptor structure suitable for subsequent molecular docking investigations.

### 4.2. Binding Pocket Identification Using Deep Learning

Automated detection of potential ligand-binding sites on the protein surface was accomplished using the deep learning-based tool UniSite (https://github.com/quanlin-wu/unisite, accessed on 19 May 2026) [31]. This model, based on a graph neural network architecture, integrates geometric and physicochemical features of the protein for accurate prediction. The specific workflow was as follows: a computational environment based on the PyTorch (Version 2.2.0) deep learning framework was set up under the Linux operating system. The input target protein PDB file underwent standardized preprocessing, including removal of crystallographic water molecules, addition of polar hydrogen atoms, and energy minimization, followed by conversion into a spatial graph data structure containing atomic three-dimensional coordinates, element types, and chemical features. The UniSite model utilized its physics-aware evolution operator to extract residue spatial neighborhood features and charge distribution patterns, performed forward inference using pre-trained weight parameters, and calculated ligand-binding probability scores for each grid point on the full protein surface. Finally, high-scoring regions were subjected to spatial clustering analysis to determine discrete pocket coordinates. Among all identified candidate pockets, we considered pocket volume, depth index, and drug score. Pockets with a drug score ≥ 0.8 and the largest volume were selected as target sites for subsequent virtual screening. These pockets typically correspond to known ATP-binding domains or allosteric regulatory sites.

### 4.3. Construction of Natural Product Compound Libraries

Screening ligand databases were obtained from the COCONUT natural product repository (https://coconut.naturalproducts.net/, accessed on 8 December 2025), which encompasses a broad collection of structurally heterogeneous natural compounds. A total of approximately 695,000 compounds were retrieved for analysis. To enhance screening selectivity and hit quality, preliminary filtering was implemented using Lipinski’s Rule of Five (RO5) in conjunction with the Quantitative Estimate of Druglikeness (QED) scoring metric. Compounds were retained when satisfying no more than one RO5 violation alongside a QED score exceeding 0.5. Furthermore, to access the distinctive chemical space of ocean-derived compounds, the marine natural product subset was separately obtained from the Comprehensive Marine Natural Products Database (CMNPD) (https://cmnpd.org/, accessed on 13 December 2025), with identical drug-likeness filtering criteria applied.

### 4.4. Deep Learning-Based High-Throughput Virtual Screening

Large-scale virtual screening was conducted using the deep learning-based molecular docking tool DiffDock. In contrast to conventional force field-driven docking programs, DiffDock adopts a generative modeling architecture grounded in diffusion processes, navigating ligand binding conformations and pose space through iterative reverse denoising operations, thereby achieving notable advances in predictive precision and computational throughput. For each protein–ligand combination, DiffDock generated multiple candidate binding poses ranked according to confidence scores reflecting the model’s assessment of pose reliability. The highest-ranked pose (Rank 1) was selected for each compound, with its associated confidence score serving as the primary evaluation criterion. Candidate compounds were subsequently prioritized through comparative binding assessment for advanced characterization.

### 4.5. Conventional Molecular Docking Validation and Interaction Analysis

To further understand the interaction mode between candidate compounds and the target protein, the selected candidates underwent cross-validation using the traditional docking software QuickVina 2 (QVina2, version 2.1). QVina2 is an improved version of AutoDock Vina (version 1.2.7), employing a more efficient search algorithm while maintaining the accuracy of the Vina scoring function. After docking, the optimal binding pose and its corresponding binding energy (kcal/mol) were extracted for each compound. More negative binding energy values indicate more stable binding. The consistency of binding modes predicted by DiffDock and QVina2 was compared, and compounds with unstable binding or significant discrepancies were excluded. Non-covalent interaction characterization was performed using the Protein–Ligand Interaction Profiler (PLIP), which generated comprehensive interaction diagrams encompassing hydrogen bonding, hydrophobic contacts, π-stacking arrangements, and salt bridge formations. Pharmacophoric features were subsequently delineated through manual interpretation of interaction analysis outcomes and rendered in PyMOL to illuminate the functional contributions of key chemical groups to target binding.

### 4.6. Molecular Dynamics Simulation Preparation

The simulation system was constructed based on the top-ranked binding pose generated by DiffDock. Protein atomic interactions were described using the AMBER99SB-ILDN force field, while ligand topology parameters were derived using Sobtop (version 1.0 dev3.2). Atomic partial charges for ligand atoms were determined through the restrained electrostatic potential (RESP) fitting approach implemented within Multiwfn [32,33]. The specific steps were as follows: first, ligand structure optimization and single-point energy calculations were performed in the ORCA (version 5.0.4) quantum chemistry program package at the B3LYP/6-31G theory level to generate an electrostatic potential file; this file was then read by Multiwfn to fit RESP charges; finally, the fitted RESP charges were integrated into the topology file generated by Sobtop. The protein–ligand complex was placed at the center of a cubic box with a minimum distance of 10 Å between the box boundary and the complex surface. The box was filled with TIP3P water molecules. To neutralize the system’s net charge, an appropriate number of Na^+^ or Cl^−^ ions were added.

### 4.7. System Energy Minimization and Pre-Production Equilibration

Resolution of steric clashes and unfavorable atomic contacts was achieved through energy minimization employing the steepest descent algorithm, with convergence defined by a maximum force threshold of 1000 kJ/(mol·nm). Thermal equilibration was subsequently carried out by incrementally raising the system temperature from 0 K to 300 K within the canonical (NVT) ensemble across 100 ps, during which protein heavy atoms were restrained with a force constant of 1000 kJ/(mol·nm^2^). Pressure equilibration was then conducted under isothermal–isobaric (NPT) conditions for an additional 100 ps, applying Berendsen or Parrinello–Rahman pressure coupling algorithms to stabilize pressure at 1 bar and temperature at 300 K until system density reached a steady state.

### 4.8. Production-Phase Molecular Dynamics Simulations

Assessment of protein–ligand complex dynamic behavior and binding conformation stability was performed through unconstrained production MD simulations spanning 200 ns, executed within the GROMACS (version 2024.4) computational platform. A 2 fs integration time step was adopted throughout, with all chemical bond lengths constrained via the LINCS algorithm. Thermal regulation and barostatic control were maintained using the v-rescale thermostat and Parrinello–Rahman barostat, respectively. A cutoff distance of 10 Å was applied to van der Waals interactions, whereas long-range electrostatic contributions were handled through the Particle Mesh Ewald (PME) algorithm. Coordinate trajectory snapshots were recorded at 10 ps intervals for downstream analysis.

### 4.9. Binding Affinity Estimation via Free Energy Calculations

Quantitative evaluation of candidate compound binding affinity toward the target protein was accomplished using the Molecular Mechanics/Generalized Born Surface Area (MM-GBSA) framework. This end-point free energy methodology estimates binding thermodynamics by averaging energy contributions across MD trajectory snapshots, offering an effective compromise between computational demand and predictive accuracy. The final 100 frames extracted from the 200 ns production trajectory via the GROMACS utility gmx trjconv were subjected to binding free energy calculation using gmx_MMPBSA. Gas-phase energy contributions, encompassing internal and van der Waals terms, were evaluated using the sander module parameterized with the AMBER99SB-ILDN force field. Solvation free energies were approximated through the Generalized Born model (igb = 5, GB-OBC1 formulation), while nonpolar solvation contributions were computed using the solvent-accessible surface area (SASA) approach.

### 4.10. Electronic Structure Characterization by DFT

Quantum-level investigation of the molecular properties and chemical reactivity of the candidate compound was performed through density functional theory (DFT) calculations, with all computations executed using the ORCA 5.0.4 program suite. Structural optimization was carried out in the gas phase without geometric constraints at the B3LYP/def2-TZVP level of theory, after which vibrational frequency calculations at the same theoretical level verified that the optimized geometry represented a true local energy minimum (absence of imaginary frequencies). Subsequent electronic structure analysis focused on characterization of the highest occupied molecular orbital (HOMO) and lowest unoccupied molecular orbital (LUMO). The HOMO energy reflects the electron-donating capacity of the molecule, whereas the LUMO energy corresponds to its electron-accepting tendency; the resulting energy difference (HOMO–LUMO gap, ΔE) constitutes a critical descriptor of molecular reactivity and chemical stability. A reduced gap value correlates with enhanced polarizability and greater susceptibility to electronic excitation, albeit potentially at the cost of diminished kinetic stability. Graphical representation of computed results, encompassing molecular electrostatic potential (MESP) maps and frontier orbital visualizations, was generated using Multiwfn 3.8_dev.

## 5. Conclusions

This study employed a systematic computational approach integrating deep learning-based virtual screening, molecular docking, interaction profiling, molecular dynamics simulations, MM-GBSA binding free energy calculations, and DFT electronic structure analysis to identify CMNPD10921 as a putative ligand of the ASK1 central regulatory region. Convergent computational evidence—including stable complex formation over 200 ns MD simulations, a favorable MM-GBSA binding free energy of −31.15 kcal/mol dominated by van der Waals interactions, and key interacting residues ILE324, THR288, and THR639—supports the potential of this marine-derived natural product as a candidate for further investigation. It must be emphasized, however, that the present study demonstrates only that CMNPD10921 can stably occupy the selected regulatory region pocket; it does not provide direct evidence of allosteric modulation of ASK1 kinase activity, and all findings remain at the computational prediction stage. Future experimental validation through biophysical binding assays, enzymatic activity measurements, cellular functional studies, and pharmacokinetic profiling is essential to establish the true biological relevance and therapeutic potential of CMNPD10921 for anti-MASH drug development.

## Figures and Tables

**Table 1:** Physicochemical properties and drug-likeness profiles of the candidate compounds identified from the integrated virtual screening.

Source	Compound ID	Molecular Formula	InChIKey	MW	AlogP	RB	HBA	HBD	TPSA	Ar. Rings	HA	QED
COCONUT	CNP0007216.1	C20H20N2O4	QQJDLARJUPMLQR-INIZCTEOSA-N	352.39	3.07	2	4	0	59.08	2	26	0.83
COCONUT	CNP0075052.1	C22H35N5O2	WZJVYCYXSHGXOG-OTWHNJEPSA-N	401.56	2.30	4	5	1	62.63	1	29	0.84
COCONUT	CNP0141324.0	C15H16N2O2S	IZNBUCGVELPOFW-UHFFFAOYSA-N	288.37	2.83	3	4	1	51.22	2	20	0.94
COCONUT	CNP0161299.2	C16H16O4	BMADVHDZKAZTNF-LBPRGKRZSA-N	272.30	2.83	2	4	2	58.92	2	20	0.88
COCONUT	CNP0370867.1	C13H12O6S	RFLGNUWEEVPNCG-ZETCQYMHSA-N	296.30	3.26	6	5	2	100.88	2	20	0.84
COCONUT	CNP0459218.1	C16H20N2O	NUSDHFWKAARFKX-HOTGVXAUSA-N	256.35	2.89	2	2	1	32.34	1	19	0.83
COCONUT	CNP0521693.0	C14H16O6	KVXHXDMSOQZQEM-UHFFFAOYSA-N	280.28	1.83	6	4	2	93.06	1	20	0.83
COCONUT	CNP0533269.1	C20H18O6	SVXYXQNNNSZDAR-RFOWDYPYSA-N	354.36	2.92	2	6	2	93.06	2	26	0.81
COCONUT	CNP0548860.0	C14H16N2O4	WMBRNCPLZVFMRQ-UHFFFAOYSA-N	276.29	1.60	4	4	1	76.82	2	20	0.92
COCONUT	CNP0555824.0	[C16H21N2O2S]+	UXBCHTZINZNVRG-UHFFFAOYSA-N	305.42	1.79	4	2	1	64.04	2	21	0.88
CMNPD	CMNPD25082	C19H22Br2O3	FDCPNRDPYOQPJY-AENFJUBXSA-N	458.19	3.93	8	3	1	46.53	0	24	0.26
CMNPD	CMNPD20811	C18H32O4	GJGSSMGEAZMVTN-FXYWYECCSA-N	312.45	3.83	14	3	3	77.76	0	22	0.34
CMNPD	CMNPD10921	C37H45NO5	PBODNUVAXAPXJH-YTOGWHTISA-N	583.77	6.59	7	6	3	98.07	3	43	0.27
CMNPD	CMNPD22038	C16H15NO3	ZJKJPVOOYVJWFU-ZBFHGGJFSA-N	269.30	1.89	2	3	2	58.56	2	20	0.87
CMNPD	CMNPD31035	C9H16Br2O4	CKSINPBEHXSBQN-UTSKPXGSSA-N	348.03	1.10	3	4	3	69.92	0	15	0.67

InChIKey, the IUPAC International Chemical Identifier hash key, which serves as a unique, database-searchable molecular fingerprint for unambiguous compound identification and cross-database deduplication; MW, Molecular weight (Da); ALogP, Calculated octanol–water partition coefficient; RB, Number of rotatable bonds; HBA, Number of hydrogen bond acceptors; HBD, Number of hydrogen bond donors; TPSA, Topological polar surface area (Å^2^); Ar. Rings, Number of aromatic rings; HA, Number of heavy (non-hydrogen) atoms; QED, Quantitative estimate of drug-likeness. ALogP was computed using the Ghose–Crippen–Viswanadhan atom-type approach. Compounds satisfying Lipinski’s rule of five (MW ≤ 500 Da, ALogP ≤ 5, HBA ≤ 10, HBD ≤ 5) and Veber’s criteria (RB ≤ 10, TPSA ≤ 140 Å^2^) are considered to possess favorable oral bioavailability profiles. QED scores were calculated according to the weighted scheme proposed by Bickerton et al., which integrates eight molecular descriptors—MW, ALogP, HBA, HBD, TPSA, RB, aromatic ring count, and the number of structural alerts—into a single composite metric reflecting overall drug-likeness. A QED value closer to 1 indicates a higher resemblance to approved oral drugs.

**Table 2:** Binding affinities of the top-ranked candidate compounds against ASK1.

Source	Compound ID	Binding Energy (kcal/mol)
COCONUT	CNP0007216.1	−7.3
COCONUT	CNP0075052.1	−8.0
COCONUT	CNP0141324.0	−6.2
COCONUT	CNP0161299.2	−7.3
COCONUT	CNP0370867.1	−6.0
COCONUT	CNP0459218.1	−6.4
COCONUT	CNP0521693.0	−6.6
COCONUT	CNP0533269.1	−7.9
COCONUT	CNP0548860.0	−6.3
COCONUT	CNP0555824.0	−7.0
CMNPD	CMNPD25082	−5.0
CMNPD	CMNPD20811	−4.9
CMNPD	CMNPD10921	−9.1
CMNPD	CMNPD22038	−6.5
CMNPD	CMNPD31035	−6.2

## Data Availability

All data supporting the findings of this study are available within the article and its Supplementary Materials. The protein structures used in this study were retrieved from the Protein Data Bank (https://www.rcsb.org/; accessed on 22 March 2026; PDB ID: pdb_00005ulm). The natural product compound libraries were obtained from the COCONUT database (https://coconut.naturalproducts.net/; accessed on 8 December 2025) and the CMNPD (https://cmnpd.org/; accessed on 13 December 2025).

## Supplementary Materials

The following supporting information can be downloaded at: https://www.mdpi.com/article/10.3390/ijms27146438/s1.

## Abbreviations

The following abbreviations are used in this manuscript:

AIDD	Artificial Intelligence-Assisted Drug Discovery
ASK1	Apoptosis Signal-Regulating Kinase 1
BE	Binding Energy
CMNPD	Comprehensive Marine Natural Products Database
COCONUT	COlleCtion of Open Natural prodUcTs
DFT	Density Functional Theory
FEL	Free Energy Landscape
FMO	Frontier Molecular Orbital
GROMACS	GROningen MAchine for Chemical Simulations
HOMO	Highest Occupied Molecular Orbital
LINCS	LINear Constraint Solver
LUMO	Lowest Unoccupied Molecular Orbital
MAPK	Mitogen-Activated Protein Kinase
MASH	Metabolic Dysfunction-Associated Steatohepatitis
MD	Molecular Dynamics
MM-GBSA	Molecular Mechanics/Generalized Born Surface Area
NASH	Nonalcoholic Steatohepatitis
NAFLD	Nonalcoholic Fatty Liver Disease
NPT	Isothermal–Isobaric Ensemble (Constant Number of Particles, Pressure, and Temperature)
NVT	Canonical Ensemble (Constant Number of Particles, Volume, and Temperature)
PCA	Principal Component Analysis
PDB	Protein Data Bank
PLIP	Protein–Ligand Interaction Profiler
PME	Particle Mesh Ewald
QED	Quantitative Estimate of Druglikeness
RESP	Restrained Electrostatic Potential
Rg	Radius of Gyration
RMSD	Root Mean Square Deviation
RMSF	Root Mean Square Fluctuation
RO5	Lipinski’s Rule of Five
SASA	Solvent-Accessible Surface Area
